# Leveraging a randomized trial to assess relationships between transeptal puncture, brain emboli, and migraine symptoms

**DOI:** 10.1016/j.hrthm.2025.06.035

**Published:** 2025-07-07

**Authors:** Adi Elias, Roderick Tung, Edward P. Gerstenfeld, Trisha F. Hue, Feng Lin, Jing Cheng, J. Peter Weiss, Wendy S. Tzou, Henry Hsia, Ashkan Ehdaie, Daniel H. Cooper, T. Jared Bunch, Jeffrey Arkles, Babak Nazer, Adam Lee, Alexios Hadjis, Duy T. Nguyen, Mihail G. Chelu, Joshua D. Moss, Jonathan C. Hsu, Miguel Valderrabano, Prashant D. Bhave, Gabrielle Montenegro, Anthony S. Kim, William P. Dillon, Gregory M. Marcus

**Affiliations:** 1 Division of Cardiology, University of California, San Francisco, San Francisco, California; 2 Division of Cardiology, University of Arizona College of Medicine – Phoenix, Phoenix, Arizona, and Banner – University Medical Center Phoenix, Phoenix, Arizona; 3 Department of Epidemiology and Biostatistics, University of California, San Francisco, San Francisco, California; 4 Oral Epidemiology & Dental Public Health, University of California, San Francisco, San Francisco, California; 5 Division of Cardiology, University of Colorado Anschutz Medical Center, Aurora, Colorado; 6 Smidt Heart Institute, Cedars-Sinai Medical Center, Los Angeles, California; 7 Division of Cardiovascular, Washington University School of Medicine, St. Louis, Missouri; 8 Division of Cardiovascular Medicine, University of Utah, Salt Lake City, Utah; 9 Division of Cardiovascular, University of Pennsylvania, Philadelphia, Pennsylvania; 10 Division of Cardiology, University of Washington, Seattle, Washington; 11 Division of Cardiology, Hôpital du Sacré-Coeur de Montréal, University of Montreal, Montreal, Quebec, Canada; 12 Department of Cardiovascular Medicine, Mayo Clinic, Rochester, Minnesota; 13 Department of Medicine – Cardiology, Cardiovascular Research Institute, Baylor College of Medicine and Texas Heart Institute at Baylor St. Luke’s Medical Center, Houston, Texas; 14 Division of Cardiology, University of California, San Diego, San Diego, California; 15 Department of Cardiology, Houston Methodist Hospital, Houston, Texas; 16 Department of Cardiovascular Medicine, Wake Forest School of Medicine, Winston-Salem, North Carolina; 17 Department of Neurology, University of California, San Francisco, San Francisco, California; 18 Department of Radiology and Biomedical Imaging, University of California, San Francisco, San Francisco, California.

**Keywords:** Transseptal puncture, Atrial septal defect, Migraine, Visual auras, Brain emboli

## Abstract

**BACKGROUND:**

Catheter ablation procedures with transseptal punctures (usually for atrial fibrillation) are often associated with migraine-related visual auras, but the mechanism remains unknown. Whether this phenomenon is mediated by the creation of an atrial septal defect from transseptal puncture or by silent acute brain emboli detected on magnetic resonance imaging related to the procedure remains to be investigated.

**OBJECTIVE:**

This study aimed to evaluate whether randomization to a transseptal puncture during catheter ablation for ventricular arrhythmias is associated with postprocedural visual auras and assess the relationship between occipital and parietal lobes acute brain emboli and migraine-related visual auras.

**METHODS:**

In the Transseptal Versus Retrograde Aortic Ventricular Entry to Reduce Systemic Emboli trial, patients undergoing catheter ablation for ventricular arrhythmias were randomized to ventricular access via transseptal puncture vs a retrograde aortic approach. All had brain magnetic resonance imaging the day after their procedure and underwent a validated migraine assessment at 1 month.

**RESULTS:**

No differences in postablation visual auras were observed between transseptal (16% of 63) and retrograde aortic approaches (14% of 57; *P* = .78). However, more participants with acute brain emboli in the occipital or parietal lobes experienced migraine-related visual auras (38% vs 11%; *P* < .01). After multivariable adjustment, the presence of acute brain emboli was associated with 12-fold greater odds of visual auras.

**CONCLUSION:**

Transseptal puncture was not associated with visual auras; however, acute brain emboli involving the visual cortex were associated with such symptoms. These data suggest that transseptal puncture is not causal in migraine-related visual auras and that postprocedure acute brain emboli are apparently not always clinically silent.

## Introduction

Catheter ablation procedures can result in brain emboli detectable by magnetic resonance imaging (MRI), which are typically described as asymptomatic or occult.^1–3^ At the same time, migraines with visual auras have been reported to occur within the few weeks after catheter ablation for atrial fibrillation (AF),^4–7^ a procedure that has been associated with those apparently clinically occult acute brain emboli.^1,2,8,9^

The pathogenesis of migraine, both in general and in the context of catheter ablation, remains incompletely understood.^4–7,10^

Interestingly, patients with migraines more often exhibit acute brain lesions detected by MRI than controls,^11–14^ but no specific temporal relationship between the development of such brain findings and a given migraine episode has been described.^15^ Indeed, the small brain emboli observed after AF ablations are often no longer apparent when brains are reimaged a few weeks later,^8,9,16–20^ suggesting that capturing precise temporal relationships may be critical. It is also important to note that patients with strokes affecting the parietal and occipital lobes may experience visual auras akin to those described with visual migraines.^21–24^

In addition, a catheter ablation procedure creates a temporary interatrial communication by the transseptal puncture (which subsequently closes),^25^ and observational studies suggest a significantly higher prevalence of congenital atrial septal defects (ASDs) or patent foramen ovales (PFOs) among those with migraine-related visual auras.^26,27^ Notably, transseptal puncture creates this iatrogenic ASD^25^ and shares the association with those symptoms.^4–7^

The potential mechanistic connection between these phenomena (if any) remains unknown, but falls into 2 broad categories: first, perhaps migraines occur owing to right-to-left shunting of some chemical substances, or “evil humor,” that bypasses normal metabolism by the lungs, leading to headaches and visual symptoms upon exposure to the brain, or these interatrial communications may lead to paradoxical brain emboli that then manifest as migraine symptoms.^27^

Although observational studies have demonstrated a reduction of visual auras and other migraine symptoms after PFO closure,^26,28,29^ randomized trials have failed to confirm a causal link.^30–32^

Unlike procedures for AF, catheter ablation of ventricular arrhythmias can be performed via either a transseptal puncture (creating a new and temporary interatrial communication) or a retrograde aortic approach (not creating such a communication).^33^ Separately, catheter ablations of ventricular arrhythmias are associated with an especially high risk of acute brain emboli, which are often believed to be asymptomatic.^3^

We recently completed the Transseptal Versus Retrograde Aortic Ventricular Entry to Reduce Systemic Emboli (TRAVERSE) trial,^34^ wherein patients with ventricular arrhythmia were randomly assigned to either a transseptal approach (which then involves the temporary ASD)^25^ or a retrograde aortic approach to what was otherwise the same catheter ablation procedure. Participants also underwent brain MRI immediately after their procedure as part of a uniformly administered study protocol, providing a unique opportunity to investigate the association between MRI-detected acute brain emboli and migraine-related visual auras and to disentangle the potential causal role of transseptal puncture in triggering these symptoms.

## Methods

TRAVERSE was a prospective, multicenter, randomized (1:1) controlled comparative trial to assess the risk of brain emboli among those randomly assigned to a transseptal puncture vs a retrograde aortic approach for catheter ablation of ventricular arrhythmias. The trial was approved by the institutional review board at each site, and all patients provided a written informed consent.

Patients aged ≥18 years scheduled for a catheter ablation procedure for a ventricular target accessible by a retrograde aortic or by transseptal approach were eligible for the study. Patients were excluded if they had a contraindication to a transseptal approach (mitral stenosis, a mechanical mitral valve, an ASD closure device, or a MitraClip or Alfieri mitral valve repair) or a retrograde aortic approach (such as caused by aortic stenosis or a mechanical aortic valve); were undergoing a planned epicardial ablation expected to require a periprocedural coronary angiogram; had a contraindication to MRI as defined by their local institution; were unable to speak, read, and write in the English language at a sixth grade level; had a life expectancy of less than 1 year; or had mental impairment that would preclude accurate assessment of neurocognitive function or prevent that patient from understanding the nature, significance, and scope of the study.

Participant interviews and medical chart reviews were used to ascertain participant demographics and medical history. The evening immediately prior to or the morning of the procedure, participants were randomly assigned in a 1:1 ratio, stratified by site in blocks of 6, to undergo either a transseptal puncture or a retrograde aortic approach to left ventricular ablation. Although operators could not be blinded, participants were not informed of their randomization assignment unless it became important to their clinical care after the procedure. Details regarding each procedure were collected.

### Migraine and visual aura ascertainment

A validated questionnaire designed to identify and characterize interim migraine symptoms was used to assess visual auras and other migraine symptoms both at baseline and prospectively during 1- and 6-month visit windows.^35^ Given evidence that visual auras in the absence of headache frequently occur in the setting of interatrial communications, a question allowing for such a presentation was added.

Given previous evidence that interatrial communication-based migraine symptoms are most often associated with visual auras,^4,6,27^ the main outcome of interest for the current analysis was the prevalence of visual aura symptoms (with or without headache symptoms) occurring between the study procedure and the 1-month visit.

### Acute brain emboli ascertainment

On postoperative day 1, participants underwent a brain MRI using a standardized protocol including diffusion-weighted sequences to identify the presence and location of acute brain emboli (Supplemental Material), which is a highly accurate method for detecting acute ischemic brain lesions and is reliable in differentiating them from chronic brain lesions.^36^ An independent neuroradiology core blinded to the randomization assignment and catheter ablation procedure details interpreted the MRIs and detailed the presence and location of new foci with reduced diffusion characteristic of new ischemic areas.

Given previous evidence that visual aura symptoms have been associated with occipital and parietal brain lesions,^21–24^ an analysis comparing the prevalence of acute brain emboli in those lobes among those with and without visual auras was conducted. Sensitivity analyses included assessing the visual cortex (occipital lobe) alone and evaluating other brain emboli locations.

### Cognitive composite score

Each participant received a complete neurocognitive function examination at screening (prestudy ablation procedure) and at the month 6 visit. Neurocognitive function testing was previously described in the main analysis of the TRAVERSE trial^34^; *z* scores of the cognitive composite score accounting for age and education level were analyzed as continuous variables (Supplemental Material).^37–39^ The *z* scores using the same neurocognitive assessment have been previously validated compared with established clinical standards.^37–39^

### Statistical analyses

Normally distributed continuous variables are presented as means and standard deviations and were compared using *t* tests, whereas continuous variables with skewed distributions are presented as medians with interquartile ranges and were compared using the Mann-Whitney test. Categorical variables were compared using Fisher’s exact or χ^2^ test as appropriate.

Two hypotheses were tested: first, we hypothesized that visual auras would be associated with acute brain emboli observed in the parietal and occipital lobes and, second, that random assignment to a transseptal puncture compared with randomization to a retrograde aortic approach would be associated with the prevalence of visual auras reported at the 1 month.

Analyses assessing as-treated groups were also performed. Given the hypothesis that a transseptal approach was the culprit for the increased risk of visual auras, the as-treated groups were divided into those who underwent *any* transseptal approach vs *only* a retrograde puncture (such that those who underwent both approaches would be considered in the transseptal approach group).

Complete case analyses were used for the main comparisons, including only participants who completed the 1-month migraine questionnaire. Multiple imputation was performed as a secondary analysis to account for missing data.^40^ A multivariable adjusted logistic regression model including patient and procedural characteristics plausibly related to the risk of visual auras and a *P* < .1 in the unadjusted analyses was conducted (variables with biologically plausible collinearity were assessed in separate models). Statistical analyses were performed using R version 3.6.1 (R Foundation for Statistical Computing, Vienna, Austria). A 2-tailed *P* < .05 was considered statistically significant. The study was approved by the University of California, San Francisco, Institutional Review Board.

## Results

Between June 2019 and February 2023, a total of 218 patients were screened for eligibility for the study. Eventually, 146 patients underwent the left ventricular endocardial ablation, with 74 patients randomly assigned to the transseptal group and 72 randomized to the retrograde aortic group (Supplemental Figure 1).

Sixty-three patients (85%) in the transseptal group and 57 patients (79%) in the retrograde group completed the 1-month migraine questionnaire at a median of 38 days (interquartile range 34–47) after the index procedure (Supplemental Table 1). Eighteen patients experienced visual auras after the procedure, as reported at the 1-month follow-up visit. The characteristics of those with and without visual auras reported at 1 month are presented in Table 1. Women and those reporting visual auras before the index procedure were more likely to experience postoperative visual auras. Migraine questionnaire completion times did not differ significantly between those with and without visual auras (Supplemental Table 2).

Baseline and procedural characteristics between the randomization groups did not differ significantly (Supplemental Table 3). In patients assigned to the transseptal group, 95% actually received it, and 96% of those randomized to the retrograde aortic group received it. No meaningful or statistically significant differences in the prevalence of postoperative visual auras or other migraine symptoms were found between the transseptal and retrograde aortic groups, whether analyzed as randomized or as treated (Table 2).

**Figure 1:**
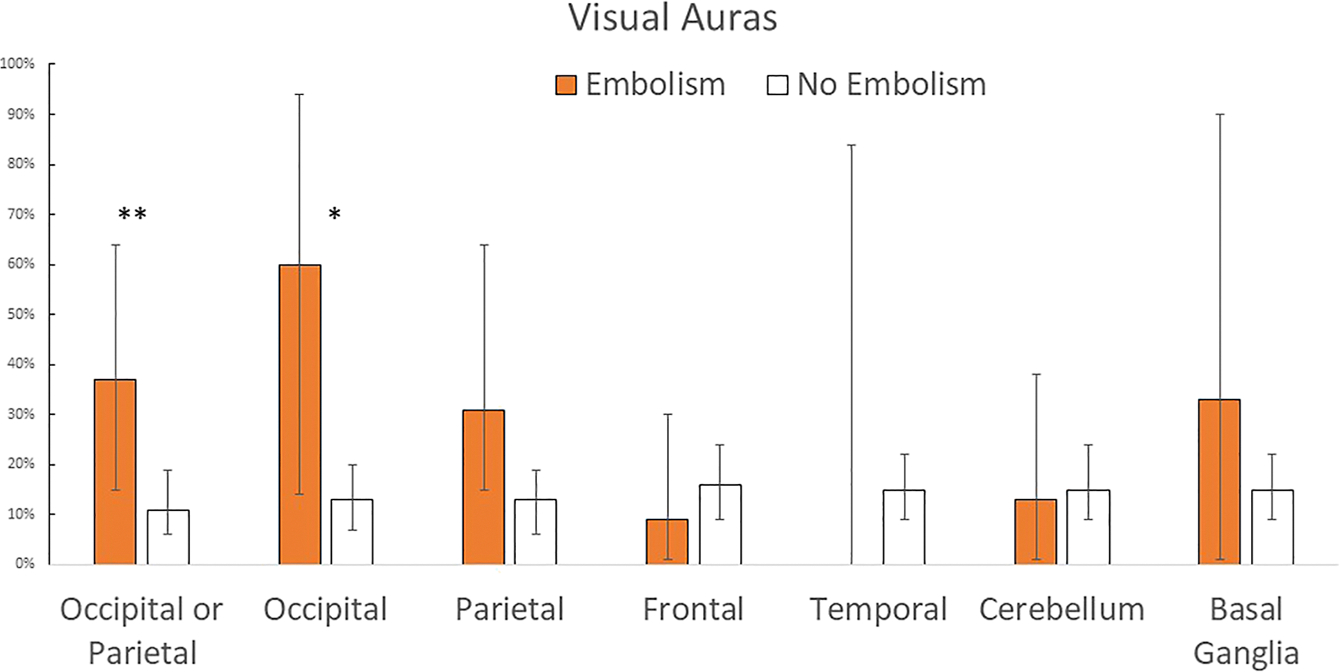
Prevalence of postoperative visual auras by acute brain emboli location. Percentage of visual auras by the presence of at least 1 embolism (*orange*) or no embolism (*white*) in each brain location. *Y error bars* denote 95% confidence intervals. *P < .05, **P < .01.

Those with postoperative day 1 occipital or parietal acute brain emboli more often experienced postoperative visual auras at 1 month (Figure 1, Supplemental Table 4). In a sensitivity analysis, those with occipital emboli also had significantly more postoperative visual auras. Moreover, the number of brain emboli in the occipital or parietal location was significantly associated with visual auras (Supplemental Table 5). Preoperative and postoperative anticoagulation patterns did not differ significantly between the randomized groups nor between those with and without brain emboli or visual auras (Supplemental Table 6).

**Figure 2:**
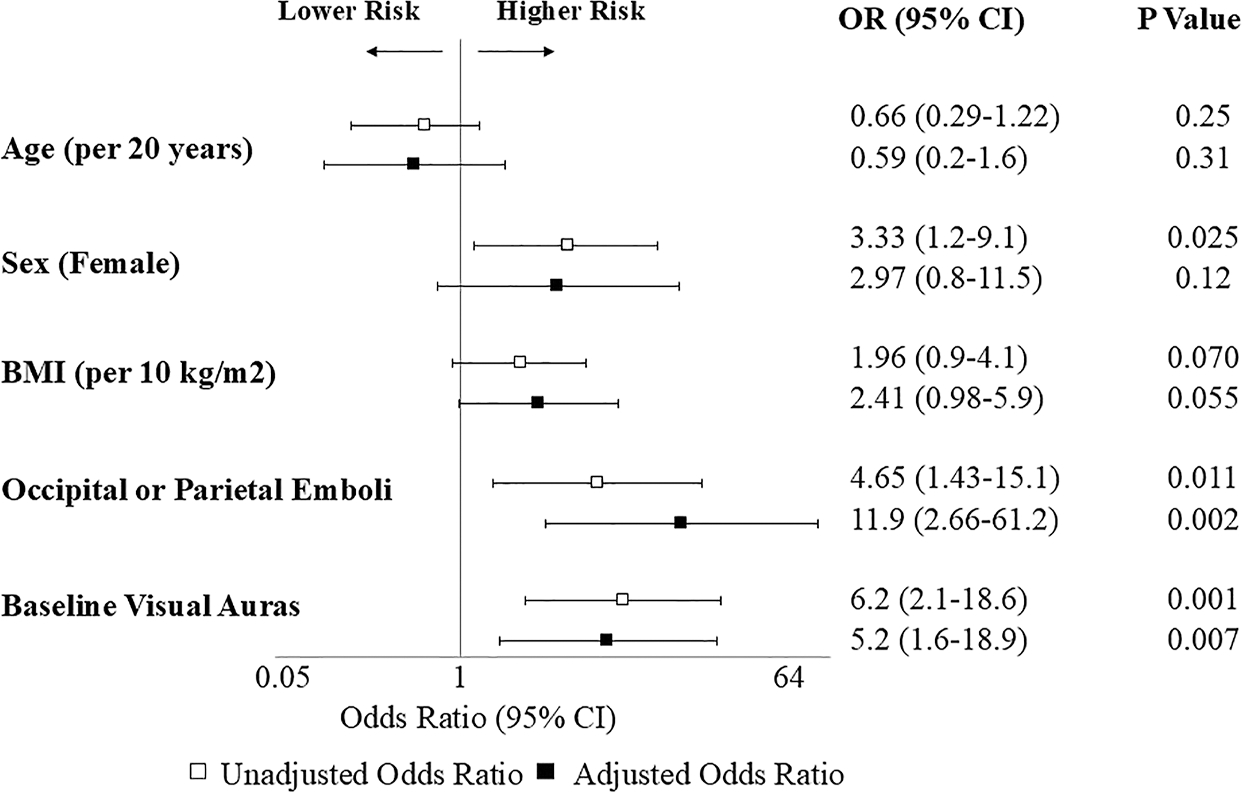
Multivariable predictors of migraine-related visual auras at 1 month. Unadjusted (*white*) and ORs adjusted for age, sex, BMI, and baseline visual auras (*black*) are shown, with Y error bars denoting 95% CIs. The *vertical line* denotes an OR of 1. BMI = body mass index; CI = confidence interval; OR = odds ratio.

In multivariable analyses, independent predictors of postoperative visual auras reported at 1 month included occipital or parietal lobe acute brain emboli and baseline visual aura symptoms (Figure 2, Supplemental Table 7). After multiple imputation to address missing data, occipital or parietal lobe acute brain emboli and baseline visual auras each remained independently associated with postoperative visual auras (Supplemental Table 8).

Neither the method of ventricular entry nor the presence of postoperative acute brain emboli in the occipital or parietal lobes was associated with either preprocedural visual auras nor visual auras assessed 6 months after the procedure (Supplemental Tables 3, 4, 9, and 10).

Although those with any migraine symptoms more often exhibited acute occipital or parietal emboli, those brain emboli were not significantly associated with headache, hypersensitivity to light or noise, or nausea and vomiting when each was examined alone (Supplemental Table 11).

The presence of brain emboli and visual auras was not associated with any significant change in the cognitive composite score (Supplemental Tables 12 and 13).

A total of 58 patients in the current analysis underwent preprocedural MRI, none of which revealed any acute emboli. A sensitivity analysis restricted to these patients was consistent with our main results; emboli in the occipital or parietal lobes were associated with a higher risk of visual auras (Supplemental Table 14), whereas the transseptal approach was not associated with these symptoms.

A sensitivity analysis restricted to patients without visual auras at baseline was consistent with our main results: emboli in the occipital or parietal lobes were associated with a higher risk of visual auras, although this did not reach statistical significance (*P* = .09) (Supplemental Table 15). The transseptal approach was not associated with these symptoms.

## Discussion

In patients with ventricular arrhythmias undergoing catheter ablation procedures, migraine-associated symptoms, including visual auras, were not associated with either random assignment to or actual receipt of a transseptal approach (resulting in a temporary interatrial communication) vs a retrograde aortic approach. However, those with migraine-related visual auras were significantly more likely to exhibit an acute brain embolism in their occipital or parietal lobes attributed to the procedure.

Small retrospective reports and anecdotal experience suggest that catheter ablation of AF, a procedure that always requires a transseptal puncture, is often associated with migraine-related visual auras shortly after the procedure.^4–7^ This fits with previous observations demonstrating a heightened prevalence of interatrial communications, such as ASDs or PFOs, among those with visual auras.^26,27^ To the best of our knowledge, the current study is the first to examine randomized assignment to the creation of a new interatrial communication vs not among patients otherwise undergoing the same procedure (in this case, catheter ablation of left ventricular arrhythmias). Our failure to demonstrate a relationship between the new creation of such an interatrial shunt and subsequent migraine symptoms is consistent with prospective studies that have generally failed to show that closure of these communications may help improve migraine symptoms.^30–32^ This lack of causal association of both PFO closure and transseptal puncture with visual auras argues against the involvement of right-to-left shunting of some chemical substances in the pathophysiology of visual auras or migraines. However, as an alternative to that potential mechanism, it remains possible that some patients with interatrial communications experience paradoxical emboli as an etiology of their symptoms—our current study was not equipped to answer that specific question.

Although iatrogenic transseptal ASDs share many characteristics with congenital PFO, they differ in origin and structure. Iatrogenic ASDs result from transseptal injury, whereas PFOs involve a flap-valve structure.^41^ The injury associated with transseptal puncture may be associated with local inflammation and thrombotic processes.^42^ Moreover, iatrogenic ASDs tend to close spontaneously over time,^25^ unlike congenital ASDs, which are long standing and persistent and may result in distinct long-term hemodynamic consequences.^43^ Therefore, caution should be exercised when generalizing the study findings to congenital ASDs and PFOs.

Although acute relationships between migraine symptoms and acute cerebral emboli have not been definitively demonstrated, more indirect evidence suggests such phenomena might be operative.^21,22,44^ In particular, those with migraine symptoms more often exhibit brain lesions and may be at a higher risk of stroke,^11–14^ and those with strokes affecting occipital and parietal lobes experience visual auras,^21–24^ akin to what has been described after catheter ablation for AF and among those with interatrial communications.^4–7^ It is worth noting that, when consecutive patients undergoing AF ablation procedures undergo brain MRI, approximately 10% to 25% exhibit acute brain emboli,^9,19,20^ but no previous study has sought to correlate those findings with subsequent migraine symptoms. Importantly, repeat brain imaging a month after catheter ablation of AF has demonstrated that these lesions can often no longer be observed.^16–18^ Because the migraine symptoms occur within the month after (and not necessarily upon awakening from the procedure), this suggests that there may be a delay between the brain injury (attributed to the procedure) and the development of the migraine symptoms. If true, brain imaging that is then done in response to those symptoms may fail to reveal evidence of brain embolism even if one was indeed the culprit (in other words, it may be too late to detect a culprit brain embolism by current brain imaging techniques after those visual aura symptoms have occurred). In the current study, brain MRI was performed on all patients within a day of the procedure, enabling time for acute brain emboli that occurred during the procedure to manifest, but potentially *before* they then developed the migraine symptoms reported at the 1-month visit. Importantly, patients were blinded to their results, and therefore, knowledge of brain emboli, especially those specifically in locations most biologically plausibly related to the main symptoms of interest, visual auras, should not have occurred. A major strength of the study was the use of a prespecified and uniform MRI protocol, which was overread by a core center blinded to the randomization group and migraine outcomes, mitigating both detection and misclassification bias.

The relationship between acute occipital and parietal emboli and migraine-related visual auras persisted in a multivariable model. Of note, the history of visual auras exhibited statistically significant relationships with 1-month visual auras. This latter observation may suggest that individuals have variable propensities to these symptoms even given the same pathophysiological injury. Importantly, no significant relationships between acute cerebral emboli and preprocedure migraine-related symptoms or 6-month symptoms were observed, suggesting a near-term temporal relationship that may also lend evidence favoring causal relationships.

In addition to providing insights that might be broadly relevant to migraine symptoms, particularly visual auras, they may also be pertinent to cerebral emboli that have been observed after numerous left-heart procedures. Indeed, in addition to occurring after AF and ventricular catheter ablation,^3,8,16,20,34^ acute cerebral emboli have been observed after common coronary angiograms and invasive aortic valve studies.^45,46^ Shared by all these reports is the assumption that they are “asymptomatic,” essentially because they do not seem to cause immediate symptoms of a stroke. However, the current study suggests they are not asymptomatic—the fact that they are associated with a perturbation of visual function may also suggest that additional less obvious (and perhaps delayed) effects may occur.

Our results should not be generalized to all patients with AF, given that pulsed field ablation technology uses significantly larger transseptal sheaths and creates larger iatrogenic ASDs,^47^ which may be associated with a higher risk of emboli and visual auras than observed in this study.

### Limitations

It is important to recognize several limitations. Approximately 20% of patients did not complete the migraine questionnaire; however, the rates of noncompletion were similar between random assignment groups. Migraine symptoms were self-reported, and although all studies of migraines rely on the same or similar methods, even a validated and prospectively obtained questionnaire might misclassify a given participant’s symptoms. These data did not include an evaluation of the specific timing of the onset (and duration) of visual aura symptoms. This current report may motivate future investigations to elucidate such specifics and therefore provide more insights into mechanisms and effects on the lives of affected ablation patients.

Although the finding may be broadly applicable to patients experiencing migraines, the study population and specific circumstances were fairly narrowly focused on those undergoing catheter ablation of ventricular arrhythmias. Because the relationship between acute brain emboli and symptoms was observational, we cannot exclude residual or unmeasured confounding. Although the assignment to undergo a transseptal or retrograde aortic approach was in fact randomized, which may arguably be used to infer causal effects, these migraine symptoms were not the primary outcome of the parent trial. Moreover, the presence of a PFO was not assessed using a consistent or uniformly applied echocardiographic protocol. However, the randomized controlled trial design is expected to balance unmeasured variables, including congenital PFO, between the randomization groups.

We cannot exclude the possibility that we failed to detect a relationship between transseptal puncture and visual auras owing to insufficient power. However, given that the numerical difference (16% in the transseptal group vs 14% in the retrograde aortic approach group) was quite small, these data suggest that any contribution purely from the transeptal to these symptoms would similarly likely be negligible.

## Conclusion

In patients with ventricular arrhythmias undergoing endocardial catheter ablation, random assignment to a transseptal approach was not associated with migraine-related visual auras. A significant association between acute occipital or parietal emboli and migraine-related visual auras was observed. These findings suggest that common migraine symptoms may be attributable to acute brain emboli and not anything caused by interatrial shunting per se. Importantly, these common postprocedure brain emboli detected on MRI are apparently not always clinically occult.

## Supplementary Material

Supplementary Data

## Figures and Tables

**Table 1:** Baseline characteristics by the presence of postoperative visual auras

Characteristic	Visual auras (n = 18)	No visual auras (n = 103)	*P* value

Age, y	60 ± 15	64 ± 12	.24
Women, n (%)	8 (44)	20 (19)	.020
Race, n (%)			.29
White	17 (94)	88 (85)	
Black	0 (0)	6 (6)	
Asian/Asian American	0 (0)	5 (5)	
Other	5.6 (0)	2 (2)	
BMI (kg/m^2^)	32 ± 10	29 ± 5	.063
Baseline visual auras, n (%)	11 (65)	23 (23)	<.001
Baseline headaches (%)	5 (28)	26 (26)	.86
Baseline visual auras with headache, n (%)	6 (33)	14 (14)	.041
Transseptal approach, n (%)	11 (61)	55 (56)	.69
Baseline beta-blocker use, n (%)	4 (27)	36 (38)	.39
Diabetes, n (%)	2 (11)	18 (18)	.50
Hypertension, n (%)	11 (61)	62 (60)	.94
Coronary artery disease, n (%)	10 (56)	45 (44)	.35
Myocardial infarction, n (%)	7 (39)	25 (24)	.19
Atrial fibrillation, n (%)	4 (22)	40 (39)	.18
Previous electrical cardioversion, n (%)	1 (25)	17 (44)	.47
Sustained VT, n (%)	4 (24)	29 (28)	.69
Polymorphic VT, n (%)	1 (6)	7 (7)	.89
Ventricular fibrillation, n (%)	1 (6)	9 (9)	.69
Aborted sudden cardiac death, n (%)	1 (6)	7 (7)	.89
Presumed PVC-induced cardiomyopathy, n (%)	7 (47)	40 (47)	.99
PVCs, n (%)	15 (88)	87 (84)	.69
Procedural characteristics			
Maximal procedural systolic BP (mm Hg)	156 (30)	157 (31)	.84
Minimal procedural systolic BP (mm Hg)	89 (18)	84 (23)	.35
Fluoroscopy time (min)	13 [5–20]	9 [1–17]	.11
Procedure time (min)	40 (6)	38 (7)	.32
Left-heart time (min)	135 (59)	150 (85)	.47
Electrical cardioversion/defibrillation, n (%)	5 (28)	23 (22)	.82
Number of ablations	7 [16–23]	8 [17–33]	.45
Maximum ablation power (W)	44 ± 7	44 ± 11	.94
Average ablation power (W)	40 ± 6	38 ± 7	.39

BMI = body mass index; BP = blood pressure; PVC = premature ventricular contraction; VT = ventricular tachycardia.

**Table 2:** Migraines symptoms at 1 month by cardiac ablation approach

Symptom	Randomization groups		
	Transseptal (n = 63)	Retrograde aortic (n = 57)	*P* value

1-mo follow-up			
Visual auras, n (%)	10 (16)	8 (14)	.78
Visual auras with headaches, n (%)	3 (5)	5 (9)	.38
Visual auras without headaches, n (%)	8 (13)	7 (12)	.95
Any headache, n (%)	12 (20)	15 (27)	.33
Headache accompanied by hypersensitivity to sound or light, n (%)	7 (12)	8 (15)	.62
Headaches with nausea or vomiting, n (%)	7 (12)	8 (15)	.59
At least 1 migraine symptom, n (%)	20 (33)	18 (33)	.98

	As-treated analysis		
	Transseptal (n = 68)	Retrograde aortic (n = 52)	*P* value

1-mo follow-up			
Visual auras, n (%)	11 (17)	7 (14)	.69
Visual auras with headaches, n (%)	3 (5)	5 (10)	.29
Visual auras without headaches, n (%)	10 (15)	5 (10)	.58
Any headache, n (%)	14 (21)	13 (26)	.55
Headache accompanied by hypersensitivity to sound or light, n (%)	8 (12)	7 (14)	.77
Headaches with nausea or vomiting, n (%)	4 (6)	3 (6)	.99
At least 1 migraine symptom, n (%)	21 (32)	17 (34)	.80

## Data Availability

Data can be shared upon reasonable request to the corresponding author.

## Appendix Supplementary data

Supplementary data associated with this article can be found in the online version at https://doi.org/10.1016/j.hrthm.2025.06.035.

## Abbreviations

AF: atrial fibrillation
ASD: atrial septal defect
MRI: magnetic resonance imaging
PFO: patent foramen ovale
TRAVERSE: Transseptal Versus Retrograde Aortic Ventricular Entry to Reduce Systemic Emboli
