# Supplementary material for: Leveraging a randomized trial to assess relationships between transeptal puncture, brain emboli, and migraine symptoms
Source: Heart Rhythm. Author manuscript; Available in PMC 2026 Jun 10. (PMC13251318; doi:10.1016/j.hrthm.2025.06.035)
Supplement: Supplementary Data [file NIHMS2175215-supplement-Supplementary_Data.docx]

**APPENDIX**

**Leveraging A Randomized Trial to Assess Relationships between Transeptal Puncture, Brain Emboli, and Migraine Symptoms**

**Migraine Questionnaire**

Baseline Questionnaire

| 1. Have you ever had recurrent headaches? | Yes/No |
| --- | --- |
| 2. Have you ever had a moderate to severe headache accompanied by nausea and/or vomiting? | Yes/No |
| 3. Have you ever had a moderate to severe headache accompanied by hypersensitivity to sound or light? | Yes/No |
| 4. Have you ever had visual disturbances (e.g. flashing lights, zigzag lines, blurred vision) lasting 5-60 minutes followed by headache? | Yes/No |
| 5. **Have you ever had visual disturbances (e.g. flashing lights, zigzag lines, blurred vision) lasting 5-60 minutes without an associated headache? | Yes/No |

Follow-up questionnaire at 1-month and 6-months

| 1. Since your last study visit, have you had recurrent headaches? | Yes/No |
| --- | --- |
| 2. Since your last study visit, have you had a moderate to severe headache accompanied by nausea and/or vomiting? | Yes/No |
| 3. Since your last study visit, have you had a moderate to severe headache accompanied by hypersensitivity to sound or light? | Yes/No |
| 4. Since your last study visit, have you had visual disturbances (e.g. flashing lights, zigzag lines, blurred vision) lasting 5-60 minutes followed by headache? | Yes/No |
| 5. **Since your last study visit, have you had visual disturbances (e.g. flashing lights, zigzag lines, blurred vision) lasting 5-60 minutes *without* an associated headache? | Yes/No |

** Prospectively (prior to study enrollment) added by the authors to the previously validated survey

**Brain magnetic resonance imaging (MRI) protocol**

All study MRIs will be read centrally by the UCSF Neuroimaging Core Facility. Upon completion of the MRIs, the clinical sites will send an electronic copy of each MRI study to the UCSF Neuroimaging Core Facility, which will be read by a single Neuroradiologist blinded to treatment assignment.

MRI studies will be obtained on a 3-Tesla unit if possible, but at the same field strength pre and post ablation. 1.5-Tesla machines are acceptable if needed, such as when the patient has an implantable cardioverter-defibrillator. Sequences will include:

1. 3D T2 weighted fluid-attenuated inversion recovery (FLAIR) obtained in the sagittal plane and reformatted into the axial (parallel to the AC-PC line) and coronal (orthogonal to axial) planes. Repetition time/echo time/inversion time = 5800 ms/117.5 ms/1560 ms, echo train length 160, matrix 256x256, slice thickness 1.1 mm, field of view 24 cm, scan time ~6.5 minutes.
2. Axial Diffusion weighted image (DWI) suggested parameters: Axial plane, repetition time/echo time = 12s/minimum, b=1000 s/mm2, 3 directions, number of excitations = 4, matrix = 112x140, slice thickness 2 mm, field of view 24 cm, scan time ~ 2.4 minutes.
3. Coronal DWI imaging, suggested parameters: Coronal plane, repetition time/echo time = 12s/minimum, b=1000 s/mm2, 3 directions, number of excitations = 4, matrix = 100x90, field of view 24 cm, scan time ~ 2.4 minutes.
4. Susceptibility weighted image: 3D axial plane. Flip angle =10, TR minimum, TE minimum msec, number of echoes=3, thickness: 2.8 mm, spaced 1.1 mm, (locations per slab 88) FOV 25.6 cm.

**Neurocognitive Function Testing**

Each participant receives a complete neurocognitive function examination at Screening (pre-study ablation procedure), and at the Month 6 Visit for assessment of the second primary study objective.

An abbreviated neurocognitive function examination was provided in special circumstances and made broadly available as a result of the COVID-19 pandemic. If a screenee indicated that he/she was not interested in participating in this trial because he/she does not want to attend an in-person pre-ablation visit and/or the study site’s institutional policies currently did not allow research study visits, then that screenee was offered an abbreviated neurocognitive function examination via a Zoom video conference to be performed at baseline and at the Month 6 visit, which included 5 of the 7 items originally included in the full TRAVERSE Testing Battery. The abbreviated neurocognitive function exam included:  Favorites (Forms A and B), Line Orientation, Favorites Delay (Forms A and B), Favorites Recognition (Forms A and B), Dot Counting (Forms A and B), and Running Dots.  These tasks measured episodic memory, working memory, and spatial cognition. The only items (Flanker and Match tests) not included in this abbreviated exam were those that are dependent on reaction time and thus require in-person administration. Studies to date have documented the feasibility of remote procedures, and have shown that remote neuropsychological testing is a valid and reliable alternative to traditional face-to-face assessment.^37–39^

Testing was conducted by a physician or research coordinator at the clinical site, trained in the TRAVERSE neurologic examination procedure and blinded to treatment assignment, if possible. Every effort was made to make the neurocognitive assessments identical, prior to the study procedure and at the follow-up visit. The test data was electronically transmitted and interpreted by the Neuropsychology Core at the UCSF CC, blinded to treatment assignment.

The full in-person neurocognitive function examination included items from the Brain Health Assessment, a validated, multi-domain testing battery developed by Dr. Katherine Possin, UCSF, which aims to detect cognitive impairment in older adults. The exam also included items from the NIH EXAMINER battery and a test of spatial working memory. The TRAVERSE Testing Battery included the following tests, administered in English or French (if preferred by the participant): Favorites (Forms A and B), Match (Forms A and B), Favorites Delay (Forms A and B), Favorites Recognition (Forms A and B), Dot Counting (Forms A and B), Flanker, Running Dots.

**Figure S1 Consort Diagram**


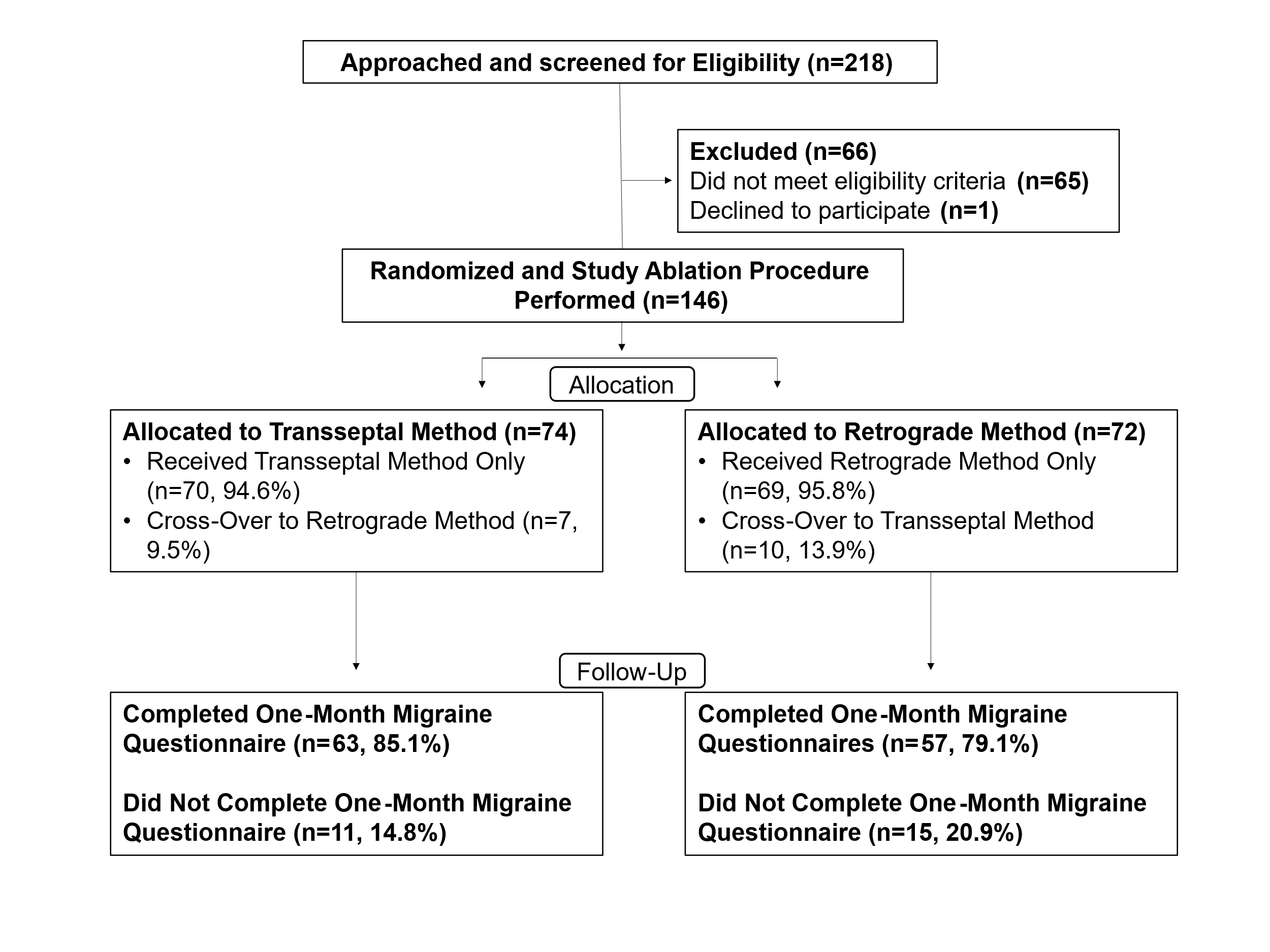


**Table S1. Distribution of Migraine Questionnaire Completion Time**

| Migraine Questionnaire Completion | Median | Mean | Range | Q1 | Q3 |
| --- | --- | --- | --- | --- | --- |
| 1 month questionnaire | 38 | 79 | 14-1177 | 34 | 47 |
| 6 months questionnaire | 200 | 260.9 | 38-1301 | 177 | 236 |

**Table S2. Distribution of Migraine Questionnaire Completion Time Stratified by Visual Auras**

| Days to Migraine Questionnaire Completion | Visual auras | No Visual Auras | P |
| --- | --- | --- | --- |
| 1 month questionnaire - median [IQR] | 37 [30, 42] | 39 [34, 47] | 0.07 |
| 6 months questionnaire - median [IQR] | 187 [169, 203] | 198 [177, 233] | 0.13 |

**Table S3. Baseline Characteristics by Cardiac Ablation Approach**

|  | Transseptal Group | Retrograde Group | p |
| --- | --- | --- | --- |
| N | 74 | 72 |  |
| Age, years | 65 ± 11 | 63 ± 13 | 0.29 |
| Female - no. (%) | 18 (24) | 12 (17) | 0.25 |
| Race - no. (%) |  |  | 0.11 |
| White | 67 (91) | 60 (83) |  |
| Black | 2 (3) | 5 (7) |  |
| Asian/Asian American | 2 (3) | 5 (7) |  |
| Other | 3 (4) | 2 (2) |  |
| BMI (kg/m^2^) | 30 ± 8 | 30 ± 7 | 0.63 |
| Baseline visual auras - no. (%) | 20 (29) | 17 (24) | 0.57 |
| Baseline headaches - no. (%) | 18 (25) | 14 (20) | 0.58 |
| Baseline visual auras with headache - no. (%) | 12 (17) | 8 (11) | 0.49 |
| Transseptal approach - no. (%) | 70 (94) | 10 (14) | <0.001 |
| Post-operative acute brain emboli - no. (%) | 19 (28) | 28 (45) | 0.035 |
| Baseline beta-blocker use - no. (%) | 25 (37) | 21 (34) | 0.78 |
| Diabetes - no. (%) | 16 (22) | 13 (18) | 0.59 |
| Hypertension - no. (%) | 46 (62) | 46 (64) | 0.83 |
| Coronary Artery Disease - no. (%) | 34 (46) | 40 (56) | 0.25 |
| Myocardial Infarction - no. (%) | 19 (26) | 25 (35) | 0.23 |
| Atrial Fibrillation - no. (%) | 30 (41) | 22 (31) | 0.23 |
| Previous Electrical Cardioversion - no. (%) | 15 (50) | 6 (29) | 0.13 |
| Sustained VT - no. (%) | 20 (27) | 23 (32) | 0.55 |
| Polymorphic VT - no. (%) | 6 (8) | 3 (4) | 0.31 |
| Ventricular Fibrillation - no. (%) | 8 (11) | 6 (8) | 0.57 |
| Aborted Sudden Cardiac Death- no. (%) | 6 (8) | 2 (3) | 0.15 |
| Presumed PVC-induced cardiomyopathy- no. (%) | 27 (47) | 27 (44) | 0.8 |
| Premature Ventricular Contractions - no. (%) | 59 (81) | 61 (85) | 0.53 |
| **Procedural Characteristics** |  |  |  |
| Maximal procedural Systolic BP (mmHg) | 160 (34) | 154 (32) | 0.30 |
| Minimim procedural Systolic BP (mmHg) | 86 (20) | 83.3 (25) | 0.43 |
| Fluoroscopy Time (minutes) | 9.5 [2.4- 21.2] | 6.6 [0.4- 11.9] | 0.053 |
| Procedure Time (minutes) | 237 (104) | 238 (116) | 0.98 |
| Left heart time (minutes) | 139 (79) | 154 (86) | 0.29 |
| Electrical cardioversion/ defibrillation – no. (%) | 19 (26) | 17 (24) | 0.57 |
| Number of ablations | 7 [14-23] | 9 [18-39] | 0.12 |
| Maximum power (watts) | 43 (12) | 43 (8) | 0.97 |
| Average power (watts) | 38 (8) | 37 (8) | 0.90 |

BMI denotes body mass index; VT denotes ventricular tachycardia; PVC denotes premature ventricular contraction

**Table S4. Visual Auras and Migraine Symptoms by the Presence of Occipital or Parietal Emboli**

|  | Occipital or Parietal Post-Operative Acute Brain Emboli | No Occipital or Parietal Post-Operative Acute Brain Emboli | P |
| --- | --- | --- | --- |
| Baseline visual auras - no. (%) | 6 (39) | 12 (24) | 0.24 |
| Visuals auras 1-month - no. (%) | 7 (38) | 12 (11) | 0.015 |
| Visuals auras with headaches - no. (%) | 3 (19) | 5 (5) | 0.071 |
| Visual auras without headaches - no. (%) | 4 (25) | 11 (11) | 0.11 |
| Any headache - no. (%) | 6 (37) | 22 (21) | 0.14 |
| Headache accompanied by hypersensitivity to sound or light - no. (%) | 1 (6) | 14 (13) | 0.69 |
| Headaches with nausea or vomiting - no. (%) | 1 (6) | 6 (6) | 1 |
| At least on migraine symptom - no. (%) | 9 (56) | 30 (29) | 0.042 |

**Table S5: Mean Number of Brain Emboli Stratified by Visual Auras**

| Emboli Location | Visual Auras | | No Visual Auras | |  |  |
| --- | --- | --- | --- | --- | --- | --- |
|  | Mean | Total Emboli | Mean | Total Emboli | Odds Ratio (95% CI) per 1 Brain Emboli Increase | P |
| Occipital or Parietal | 0.43 | 7 | 0.13 | 13 | 2.77 (1.11-6.87) | 0.02 |
| Occipital | 0.16 | 3 | 0.02 | 2 | 10.1 (1.55-81.63) | 0.015 |
| Parietal | 0.22 | 4 | 0.10 | 11 | 1.90 (0.56-5.47) | 0.24 |
| Frontal | 0.16 | 3 | 0.24 | 25 | 0.74 (0.19-1.86) | 0.58 |
| Temporal | 0 | 0 | 0.02 | 2 | *NA** | *NA** |
| Cerebellum | 0.11 | 2 | 0.14 | 14 | 0.79 (0.11-3.22) | 0.77 |
| Basal Ganglia | 0.05 | 1 | 0.02 | 2 | 2.97 (0.13-32.7) | 0.38 |
| All Brain Locations | 1.12 | 18 | 0.75 | 75 | 1.11 (0.81-1.42) | 0.43 |

*Not applicable (NA) indicates estimates could not be reliably calculated due to model instability.

**Table S6. Peri-Operative Anticoagulation**

|  | ***Randomization Groups*** | |  |
| --- | --- | --- | --- |
|  | Transseptal | Retrograde Aortic | P |
| **Pre-operative anticoagulation** |  |  |  |
| NOACs - no. (%) | 18 (24) | 21 (29) | 0.50 |
| Continuation - no. (%) | 9 (50) | 11 (52) | 0.88 |
| Interruption - no. (%) | 9 (50) | 10 (48) |  |
| Warfarin - no. (%) | 2 (2.7) | 3 (4.1) | 0.63 |
| INR < 2 - no. (%) | 1 (50) | 2 (66) | 0.54 |
| **Post-operative anticoagulation** |  |  |  |
| Heparin or LMWH - no. (%) | 3 (4) | 2 (2.7) | 0.36 |
| Initiation – hours after the procedure – median [IQR] | 3 [2, 4.5] | 5.5 [5.25, 5.75] | 0.55 |
| NOACs - no. (%) | 36 (48.6) | 33 (45.8) | 0.54 |
| Initiation – hours after the procedure – median [IQR] | 6 [4, 12.5] | 12 [5.75, 23.2] | 0.07 |
|  | ***Post-Operative Brain Emboli*** | |  |
|  | Brain Emboli | No Brain Emboli | P |
| **Pre-operative anticoagulation** |  |  |  |
| NOACs - no. (%) | 15 (32) | 21 (24) | 0.29 |
| Continuation - no. (%) | 7 (47) | 11 (52) | 0.73 |
| Interruption - no. (%) | 8 (53) | 10 (48) |  |
| Warfarin - no. (%) | 1 (2) | 4 (5) | 0.47 |
| INR < 2 - no. (%) | 0 (0) | 3 (3) | 0.20 |
| **Post-operative anticoagulation** |  |  |  |
| Heparin or LMWH - no. (%) | 1 (2) | 2 (2) | 0.25 |
| Initiation – hours after the procedure – median [IQR] | 6 [6, 6] | 3.5 [2.25, 4.75] | 0.8 |
| NOACs - no. (%) | 22 (46) | 39 (44) | 0.33 |
| Initiation – hours after the procedure – median [IQR] | 6 [3, 24] | 9 [5, 16] | 0.81 |
|  | ***1-Month Visual Auras*** | |  |
|  | Visual Auras | No Visual Auras | P |
| **Pre-operative anticoagulation** |  |  |  |
| NOACs - no. (%) | 4 (22) | 31 (30) |  |
| Continuation - no. (%) | 3 (75) | 15 (48) | 0.31 |
| Interruption - no. (%) | 1 (25) | 16 (51) |  |
| Warfarin - no. (%) | 1 (5) | 3 (3) | 0.56 |
| INR < 2 - no. (%) | 0 | 2 (2) | 0.55 |
| **Post-operative anticoagulation** |  |  |  |
| Heparin or LMWH - no. (%) | 0 (0) | 2 (2) | 0.7 |
| Initiation – hours after the procedure – median [IQR] | - | 6 [6, 6] | - |
| NOACs - no. (%) | 7 (39) | 51 (49) | 0.55 |
| Initiation – hours after the procedure – median [IQR] | 8 [4.5, 18] | 7 [4, 16] | 0.77 |

NOACs denotes novel oral anticoagulants, LMWH denotes Low Molecular Weight Heparin

**Table S7. Unadjusted and Multivariable Models for Predictors of Visual Auras at 1-Month Following Catheter Ablation**

| Variable | Unadjusted |  | Multivariable (Model 1) Occipital or Parietal |  | Multivariable (Model 2)  Occipital |  | Multivariable  (Model 3)  All Location |  |
| --- | --- | --- | --- | --- | --- | --- | --- | --- |
|  | Odds Ratio (95% CI) | P | Odds Ratio (95% CI) | P | Odds Ratio (95% CI) | P | Odds Ratio (95% CI) | P |
| Age (per year) | 0.98 (0.94-1.01) | 0.25 | 0.97 (0.93-1.03) | 0.306 | 0.98 (0.94- 1.04) | 0.64 | 0.97 (0.92-1.026) | 0.31 |
| Sex (Female) | 3.33 (1.16-9.1) | 0.025 | 2.97 (0.77-11.5) | 0.12 | 2.86 (0.76-10.5) | 0.12 | 1.54 (0.38-5.64) | 0.52 |
| BMI (kg/m^2^) | 1.07 (0.99-1.15) | 0.074 | 1.1 (0.99-1.19) | 0.055 | 1.076 (0.99-1.18) | 0.095 | 1.043 (0.95-  1.15) | 0.35 |
| Post-operative acute brain emboli | 2.57 (0.88-7.51) | 0.084 |  |  | - | - | 3.102 (0.906-11.701) | 0.077 |
| Occipital lobe emboli | 10.1 (1.56-65.5) | 0.015 |  |  | 15.81 (1.99-152.8) | 0.009 | - |  |
| Occipital or Parietal emboli | 4.65 (1.43-15.09) | 0.011 | 11.9 (2.57-55.8) | 0.002 |  |  |  |  |
| Transseptal Approach | 1.15 (0.42-3.16) | 0.78 |  |  | - | - | - |  |
| Hemoglobin (g/dL) | 1.01 (0.72-1.38) | 0.99 |  |  | - | - | - |  |
| Baseline headache | 1.11 (0.36-3.41) | 0.86 |  |  | - | - | - |  |
| Baseline visuals auras | 6.217 (2.07-18.64) | <0.001 | 5.23 (1.54-17.7) | 0.008 | 4.4 (1.36-15.51) | 0.014 | 4.36 (1.35- 14.93) | 0.014 |
| Baseline beta-blocker use | 0.59 (0.17-1.98) | 0.39 |  |  | - | - | - |  |
| Diabetes | 0.59 (0.12-2.8) | 0.51 |  |  | - | - | - |  |
| Hypertension | 1.04 (0.37-2.9) | 0.94 |  |  | - | - | - |  |
| Coronary artery disease | 1.61 (0.59-4.41) | 0.35 |  |  | - | - | - |  |
| Myocardial infarction | 1.99 (0.7-5.67) | 0.2 |  |  | - | - | - |  |
| Atrial fibrillation | 0.45 (0.14-1.46) | 0.19 |  |  | - | - | - |  |
| Electrical cardioversion | 0.43 (0.04-4.52) | 0.48 |  |  | - | - | - |  |
| Monomorphic VT | 0.79 (0.24-2.61) | 0.69 |  |  | - | - | - |  |
| Polymorphic VT | 0.86 (0.1-7.44) | 0.89 |  |  | - | - | - |  |
| Ventricular fibrillation | 0.65 (0.08-5.45) | 0.69 |  |  | - | - | - |  |
| Aborted sudden death | 0.86 (0.1-7.44) | 0.89 |  |  | - | - | - |  |
| PVCs | 1.38 (0.29-6.62) | 0.69 |  |  | - | - | - |  |
| PVC-induced cardiomyopathy | 1.01 (0.33-3.02) | 0.99 |  |  | - | - | - |  |

Variables with P < 0.1 were entered to the mode, age and sex forced into the model.

BMI denotes body mass index; VT denotes ventricular tachycardia; PVC denotes premature ventricular contraction

**Table S8. Multivariable Models with Multiple Imputation of Missing Values for Predictors of Visual Auras at 1-Month Following Catheter Ablation**

|  | Imputed Multivariable Model 1 |  | Imputed Multivariable Model 2 |  | Imputed Multivariable Model 3 |  |
| --- | --- | --- | --- | --- | --- | --- |
|  | Odds Ratio (95% CI) | P | Odds Ratio (95% CI) | P | Odds Ratio (95% CI) | P |
| Age (per year) | 0.97 (0.93-1.02) | 0.28 | 0.99 (0.94-1.04) | 0.6 | 0.98 (0.93-1.03) | 0.33 |
| Sex (Female) | 2.63 (0.63-11.11) | 0.18 | 2.77 (0.787-10) | 0.11 | 1.47 (0.4-5.26) | 0.56 |
| BMI | 1.08 (0.95-1.23) | 0.21 | 1.05 (0.98- 1.13) | 0.15 | 1.04 (0.95-1.14) | 0.34 |
| Post-operative acute brain emboli | - |  | - | - | 2.59 (0.75-8.91) | 0.12 |
| Occipital or Parietal emboli | 8.5 (1.8-40.6) | 0.011 | - | - | - | - |
| Occipital lobe emboli | - | - | 14.47 (1.79 -117.12) | 0.012 | - | - |
| Baseline visuals auras | 4.19 (1.17-14.95) | 0.032 | 3.83 (1.00-14.76) | 0.051 | 3.62 (1.06-12.28) | 0.041 |

BMI denotes body mass index

**Table S9. Migraines Symptoms at 6-Months by Cardiac Ablation Approach**

|  | ***Randomization Groups*** | | |
| --- | --- | --- | --- |
|  | Transseptal | Retrograde Aortic | p |
| N | 63 | 57 |  |
| ***6-Months Follow-up*** |  |  |  |
| Visual auras - no. (%) | 12 (21) | 8 (17) | 0.64 |
| Visual auras with headaches - no. (%) | 3 (5) | 5 (11) | 0.29 |
| Visual auras without headaches - no. (%) | 11 (19) | 5 (11) | 0.24 |
| Any headaches - no. (%) | 17 (30) | 13 (28) | 0.86 |
| Headache accompanied by hypersensitivity to sound or light - no. (%) | 8 (14) | 5 (11) | 0.63 |
| Headaches with nausea or vomiting - no. (%) | 3 (5) | 2 (4) | 0.83 |
| At least on migraine symptom - no. (%) | 24 (38) | 15 (30) | 0.37 |
|  | ***As-Treated-Analysis*** | | |
|  | Transseptal | Retrograde Aortic | p |
| n | 68 | 47 |  |
| ***6-Months Follow-up*** |  |  |  |
| Visuals auras - no. (%) | 15 (22) | 6 (13) | 0.24 |
| Visual auras with headaches - no. (%) | 5 (7) | 3 (7) | 0.89 |
| Visual auras without headaches - no. (%) | 13 (19) | 4 (9) | 0.14 |
| Any headaches - no. (%) | 19 (28) | 11 (24) | 0.68 |
| Headache accompanied by hypersensitivity to sound or light- no. (%) | 9 (13) | 4 (9) | 0.48 |
| Headache with nausea and vomiting - no. (%) | 3 (4) | 3 (7) | 0.6 |
| At least on migraine symptom - no. (%) | 27 (40) | 12 (27) | 0.15 |

**Table S10. Unadjusted and Multivariable Models for Predictors of Visual Auras at 6 Month Following Catheter Ablation**

| Variable | Unadjusted |  | Multivariable Model 1  Occipital or Parietal |  | Multivariable Model 2  Occipital |  |
| --- | --- | --- | --- | --- | --- | --- |
|  | Odds Ratio (95% CI) | P | Odds Ratio (95% CI) | P | Odds Ratio (95% CI) | P |
| Age (per year) | 1.001 (0.96-1.04) | 0.928 | 1.001 (0.96-1.05) | 0.95 | 1.001 (0.96 - 1.04) | 1 |
| Sex (Female) | 1.49 (0.53-4.16) | 0.441 | 0.93 (0.26-3.03) (0.33-3.88) | 0.905 | 0.92 (0.25-3.03) | 0.89 |
| BMI (kg/m^2^) | 1.02 (0.97-1.07) | 0.454 | - | - | - | - |
| Creatinine (mg/dL) | 0.52 (0.1-2.77) | 0.443 |  |  |  |  |
| Post-operative acute brain emboli | 1.2 (0.46-3.14) | 0.711 | - | - | - | - |
| Occipital lobe emboli | 1.5 (0.16-14.08) | 0.723 | - | - | 0.504 (0.022-4.57) | 0.57 |
| Occipital or Parietal emboli | 1.21 (0.32-4.58) | 0.778 | 0.66 (0.12-2.86) | 0.601 | - | - |
| Transseptal Approach | 1.35 (0.53-3.44) | 0.523 | - | - | - | - |
| Hemoglobin (g/dL) | 1.005 (0.75-1.34) | 0.97 |  | - | - | - |
| Baseline Headache | 1.98 (0.75-5.2) | 0.165 | - | - | - | - |
| Baseline Visual Auras | 9.41 (3.44,25.71) | <0.001 | 8.87 (3.13-27.59) | <0.001 | 8.83 (3.138- 27.31) | <0.001 |
| Baseline beta-blocker use | 1.38 (0.51-3.71) | 0.521 | - | - | - | - |
| Diabetes | 0.63 (0.17-2.3) | 0.485 | - | - | - | - |
| Hypertension | 1.13 (0.44-2.88) | 0.799 | - | - | - | - |
| Coronary artery disease | 1.06 (0.43-2.63) | 0.894 | - | - | - | - |
| Myocardial infarction | 0.91 (0.33,2.5) | 0.851 | - | - | - | - |
| Atrial fibrillation | 1.1 (0.43-2.83) | 0.837 | - | - | - | - |
| Electrical cardioversion | 2.81 (0.59-13.37) | 0.194 | - | - | - | - |
| Monomorphic VT | 1.21 (0.45-3.2) | 0.707 | - | - | - | - |
| Polymorphic VT | 0.72 (0.09-6.06) | 0.763 | - | - | - | - |
| Ventricular fibrillation | 0.97 (0.2-4.65) | 0.966 | - | - | - | - |
| Aborted sudden death | 2.05 (0.39-10.87) | 0.399 | - | - | - | - |
| PVCs | 0.66 (0.22-1.99) | 0.461 | - | - | - | - |
| PVC-induced cardiomyopathy | 1.55 (0.56-4.33) | 0.403 | - | - | - | - |

Variables with P < 0.1 were entered to the mode, age and sex forced into the model.

BMI denotes body mass index; VT denotes ventricular tachycardia; PVC denotes premature ventricular contraction

**Table S11. Unadjusted Models for Predictors of Other Migraine symptoms at 1-Month Following Catheter Ablation**

|  | Headaches |  | Hypersensitivity to Light or Sound |  | Accompanying Nausea |  | Any Migraine Symptom |  |
| --- | --- | --- | --- | --- | --- | --- | --- | --- |
| Post-operative emboli location | Odds Ratio (95% CI) | P | Odds Ratio (95% CI) | P | Odds Ratio (95% CI) | P | Odds Ratio (95% CI) | P |
| All lobes | 2.31 (0.37-14.5) | 0.37 | 0.86 (0.15-4.92) | 0.86 | 0.86 (0.15-4.92) | 0.86 | 1.63 (0.73,3.66) | 0.23 |
| Occipital lobe | 2.31 (0.37-14.5) | 0.37 | 0 (0-100) | 0.99 | 0 (0,100) | 0.99 | 9.26 (1-85.82) | 0.051 |
| Occipital or Parietal lobes | 2.26 (0.74-6.91) | 0.15 | 0.43 (0.05-3.54) | 0.44 | 1.1 (0.12,9.78) | 0.93 | 3.21 (1.1,9.41) | 0.03 |

**Table S12. Cognitive Composite Score Stratified by Post-Operative Brain Emboli**

| **Neurocognitive Score** | Brain Emboli | No Brain Emboli | P |
| --- | --- | --- | --- |
| **Baseline** - median [IQR] | -1.02 [-1.89, -0.21] | -1.03 [-1.98, -0.28] | 0.90 |
| **6 months** - median [IQR] | -0.14 [-0.88 0.59] | -0.54 [-1.70, 0.30] | 0.23 |
| **Baseline-6 months difference** - median [IQR] | 0.41 [0.23, 0.74] | 0.44 [-0.27, 1.17] | 0.69 |

Z scores of the cognitive composite score accounting for age and education level.

**Table S13. Cognitive Composite Z Score Stratified by 1-Month Visual Auras**

| **Neurocognitive Score** | Visual Auras | No Visual Auras | P |
| --- | --- | --- | --- |
| **Baseline** - median [IQR] | -0.42 [-1.31, 0.06] | -0.99 [-1.98, -0.28] | 0.15 |
| **6 months** - median [IQR] | -0.04 [-1.39, 1.1] | -0.25 [-1.31, 0.41] | 0.63 |
| **Baseline-6 months difference** - median [IQR] | 0.35 [0.29, 0.64] | 0.55 [-0.24, 0.97] | 0.68 |

Z scores of the cognitive composite score accounting for age and education level.

**Table S14. Visual Auras Symptoms at 1-Month Restricted to Participants with Pre-Operative MRI**

|  | ***Presence of Occipital or Parietal Emboli*** | |  |
| --- | --- | --- | --- |
|  | Occipital or Parietal Lobe Emboli | No Occipital or Parietal Lobe Emboli | P |
| N | 8 | 50 |  |
| Visual auras | 4 (50) | 7 (14) | 0.015 |
|  | ***Randomization Groups*** | |  |
|  | Transseptal | Retrograde Aortic | P |
| N | 28 | 26 |  |
| Visual auras | 6 (21) | 5 (19) | 0.84 |

**Table S15. Visual Auras Symptoms at 1-Month Restricted to Participants without Baseline Visual Auras**

|  | ***Presence of Occipital or Parietal Emboli*** | |  |
| --- | --- | --- | --- |
|  | Occipital or Parietal Lobe Emboli | No Occipital or Parietal Lobe Emboli | P |
| N | 10 | 74 |  |
| Visual auras | 2 (20) | 4 (5.4) | 0.09 |
|  | ***Randomization Groups*** | |  |
|  | Transseptal | Retrograde Aortic | P |
| N | 39 | 41 |  |
| Visual auras | 2 (5.1) | 4 (9.8) | 0.43 |
